# The Predominant Role of Arrestin3 in General GPCR Desensitization in Platelets

**DOI:** 10.3390/jcm10204743

**Published:** 2021-10-15

**Authors:** Preeti Kumari Chaudhary, Sanggu Kim, Soochong Kim

**Affiliations:** Laboratory of Veterinary Pathology and Platelet Signaling, College of Veterinary Medicine, Chungbuk National University, Cheongju 28644, Korea; chaudharypreety11@gmail.com (P.K.C.); tkdrnfld@naver.com (S.K.)

**Keywords:** arrestin2, arrestin3, GPCR, desensitization, platelets

## Abstract

Arrestins in concert with GPCR kinases (GRKs) function in G protein-coupled receptor (GPCR) desensitization in various cells. Therefore, we characterized the functional differences of arrestin3 versus arrestin2 in the regulation of GPCR signaling and its desensitization in platelets using mice lacking arrestin3 and arrestin2. In contrast to arrestin2, platelet aggregation and dense granule secretion induced by 2-MeSADP, U46619, thrombin, and AYPGKF were significantly potentiated in arrestin3-deficient platelets compared to wild-type (WT) platelets, while non-GPCR agonist CRP-induced platelet aggregation and secretion were not affected. Surprisingly, in contrast to GRK6, platelet aggregation induced by the co-stimulation of serotonin and epinephrine was significantly potentiated in arrestin3-deficient platelets, suggesting the central role of arrestin3 in general GPCR desensitization in platelets. In addition, the second challenge of ADP and AYPGKF restored platelet aggregation in arrestin3-deficient platelets but failed to do so in WT and arrestin2-deficient platelets, confirming that arrestin3 contributes to GPCR desensitization. Furthermore, ADP- and AYPGKF-induced Akt and ERK phosphorylation were significantly increased in arrestin3-deficient platelets. Finally, we found that arrestin3 is critical for thrombus formation in vivo. In conclusion, arrestin3, not arrestin2, plays a central role in the regulation of platelet functional responses and thrombus formation through general GPCR desensitization in platelets.

## 1. Introduction

Platelets have been established as a critical player in hemostasis and thrombosis through their activation via different signaling pathways both in humans and animals. Initially, platelets are activated by a contact-dependent pathway via collagen-induced glycoprotein VI (GPVI) signaling or vWF-induced GPIb-IX-V signaling upon the exposure of the sub-endothelial matrix [1,2]. The activation further results in the release (adenosine diphosphate (ADP), epinephrine, and serotonin), generation (thromboxane A_2_ (TxA_2_)), and exposure (collagen) of agonists, which in turn can cross-talk via their respective receptor-specific platelet activation signaling pathways and culminate into shared signaling events stimulating the platelet’s shape change, granule content release, and activation of integrin αIIbβ_3_, ultimately activating “inside-out” signaling resulting in adhesion and aggregation of the platelets. Inside-out signaling further catalyzes “outside-in” signaling and leads to platelet spreading, increased granule secretion, platelet adhesion and aggregation stability, and clot retraction [3].

One of the major mechanisms of platelet activation occurs through the stimulation of receptors on the cell surface belonging to the GPCR family. Recent studies have shed light on the roles of various agonists, including thrombin, ADP, TxA_2_, serotonin, and epinephrine, and their interaction with their respective GPCRs and correlative G proteins in the regulation of events involved in platelet activation [4]. Platelet activation triggered by GPCRs comprises a succession of quick positive feedback loops that greatly amplify initial activation signals and allow for strong platelet recruitment and thrombus stability. However, there must be a control mechanism to stop or arrest the continuous activation and signaling of platelets. The uninterrupted activation of these receptors may cause a shift in the functional responses of platelets. Several mechanisms prevent the hyper-activation of GPCR signaling, among which the involvement of arrestins along with GRKs has recently been shown as the most important mechanism for turning off the growing number of GPCR-mediated signaling transduction pathways in various cells. These are the only two protein families besides heterotrimeric G proteins that have shown the capacity to interplay with the activated conformation of 7-transmembrane (7-TM) receptors, and they are considered as the key modulators of GPCR phosphorylation, desensitization, intracellular trafficking, and re-sensitization [5,6,7].

GRKs initiate the kinase-dependent homologous desensitization of GPCRs by phosphorylating the activated GPCRs. Then, arrestins, a small family of soluble proteins located in the cytoplasm, bind specifically with the GRK-phosphorylated GPCRs, block further G protein coupling, and terminate intracellular signaling by targeting the receptor–arrestin complex to clathrin-coated pits where the receptors are internalized [8,9,10]. There are four subtypes of the arrestin family, among which arrestin1 and arrestin4 (visual arrestins) are confined to rod and cone cells, respectively, while arrestin2 and arrestin3 (non-visual arrestins, also known as β-arrestin1 and β-arrestin2, respectively) are expressed ubiquitously together with platelets [11]. Structurally, arrestin isoforms are divided into two domains, N-terminal and C-terminal domains, which are 78 percent similar to the majority of the coding changes that occur at the C terminus of the protein [12]. The activation of arrestin has long been linked to the binding of the receptor’s phosphorylated cytoplasmic tail. Arrestin–receptor interaction causes a global conformational shift, releasing the C-terminal tail of arrestin that contains the clathrin and AP2 binding sites needed for the internalization of the receptor. Very recently, it has been demonstrated that the receptor core also participates in arrestin activation [13]. It has been shown that arrestin2/arrestin3 double knockout (KO) mice die in utero, while arrestin2 and arrestin3 KO mice are viable [14,15,16]. Arrestin isoforms may substitute each other functionally to some degree. It has been demonstrated that either arrestin isoform is capable of angiotensin receptor subtype 1a (AT1AR) second messenger desensitization, and the both can be functionally substituted; however, only arrestin2 can desensitize phosphoinositide turnover for protease-activated receptor-1 (PAR1) [17], while arrestin3 is required for the desensitization of second messenger generation and internalization of the class A prototype (e.g., β2-adrenergic receptor) [18,19]. P2Y_12_ receptor desensitization and internalization in 1321N1 cells are mediated by arrestins [20,21]. In addition, it has been demonstrated that arrestin2 and arrestin3 regulate T Prostanoid receptor β (TPβ) internalization in human embryonic kidney 293 (HEK293) cells [22]. These reports clearly demonstrate the critical role of arrestins in desensitization mechanisms in GPCR signaling in other cells.

Despite the importance of arrestins in GPCR-mediated signaling, the functional role of arrestins in platelet activation, as well as their underlying mechanism, has yet to be established. It has been reported that in platelets, arrestin2 modulates PAR4 and ADP receptor signaling in distinct ways [23]. By contrast, Schaff et al. have shown that platelet GPCR activation is not mediated by arrestin2 or arrestin3 and that arrestin2, but not arrestin3, has a role in prothrombotic function in vivo likely via the promotion of phosphoinositide 3-kinase (PI3K) activity [23,24]. Meanwhile, a recent study has shown that arrestin3 negatively regulates PAR4 and P2Y_12_ receptor signaling in platelets [25]. A few cell line studies have also suggested a role for arrestins in turning off GPCR signaling in platelets [20,21,26]. The functional differences of each arrestin isoform in the control of GPCR signaling and the molecular basis of GPCR desensitization in platelets are not clear. Understanding the fundamental mechanisms underlying the GPCR-mediated platelet responses can enhance our understanding of novel regulatory mechanisms regulating platelet function.

In this study, using arrestin2 and arrestin3 −/− mice, we evaluated the functional role of arrestin isoforms and their molecular basis of regulation of GPCR desensitization in platelets. We show that arrestin3, but not arrestin2, potentiates platelet aggregation and secretion induced by agonists such as ADP, AYPGKF, thrombin, and U46619. The co-stimulation of serotonin and epinephrine potentiates platelet aggregation in arrestin3-deficient platelets, demonstrating that arrestin3 plays a central role in regulating general GPCR signaling. We further show that arrestin3 regulates ADP and PAR4 receptor desensitization resulting in the regulation of G_q_- and G_i_-mediated signaling in platelets. Arrestin3 also has a role in thrombus development in vivo. In conclusion, arrestin3 is critical for the regulation of platelet function via general GPCR desensitization.

## 2. Materials and Methods

### 2.1. Materials

2-MeSADP, U46619, ADP, thrombin, serotonin, epinephrine, apyrase (type V), prostaglandin E_1_ (PGE_1_), sodium citrate, and ASA were bought from Sigma (St. Louis, MO, USA). Dr. Richard Farndale of the University of Cambridge provided CRP. AYPGKF was from Invitrogen (Carlsbad, CA, USA). Anti-phospho-Akt (Ser^473^), anti-phospho-ERK (Thr202/Tyr204), anti-Akt, anti-ERK, and anti-β-actin antibodies were from Cell Signaling Technology (Beverly, MA, USA). Horseradish peroxidase-labeled secondary antibody was bought from Santa Cruz Biotechnology (Santa Cruz, CA, USA). All additional chemicals were of reagent grade.

### 2.2. Animals

Dr. Walter Koch (Temple University, Philadelphia, PA, USA) provided arrestin2 and arrestin3 −/− mice.

### 2.3. Isolation of Mouse Platelets

Platelets were isolated as previously described [27] using blood from an equal number of male and female mice. To obtain platelet-rich plasma (PRP), whole blood was collected in the presence of sodium citrate and centrifuged at 100× *g* for 10 min at room temperature (RT). For aspirin treatment, the PRP was treated with 1 mM acetylsalicylic acid for 30 min at 37 °C. The platelets were pelleted after centrifugation at 400× *g* for 10 min, and the pellets were re-suspended in Tyrode’s buffer (pH 7.4) comprising 0.05 units/mL of apyrase, and platelets were adjusted to 2 × 10^8^ cells/mL.

### 2.4. Platelet Aggregation and Dense Granule Secretion

As described previously [27], agonist-induced platelet aggregation and secretion were assessed using a lumi-aggregometer (Chrono-Log, Havertown, PA, USA) at 37 °C under stirring conditions (900 rpm). The release of ATP from platelets was measured to assess platelet dense granule secretion by adding luciferin/luciferase reagent.

### 2.5. Immuno-Blotting

Washed platelets were activated with various agonists, and phosphorylations were quantified as described previously [28]. Platelets were stimulated with 2-MeSADP or AYPGKF, and platelet lysates were probed with anti-phospho-Akt (Ser473), anti-phospho-ERK (Thr202/Tyr204), or anti-β-actin antibodies. The Fuji-Film Luminescent Image Analyzer (LAS-3000 CH, Tokyo, Japan) was used to detect chemiluminescence.

### 2.6. In Vivo Thrombosis Model Using FeCl_3_-Induced Carotid Artery Injury

Adult mice aged 10–12 weeks were anesthetized, and FeCl_3_-induced thrombosis was measured as described previously [29]. The carotid artery was injured for 90 s with 5% FeCl_3_, and blood flow through the carotid artery was recorded for 30 min with a Doppler flow probe. The initial time to occlusion due to thrombus development and the stability of thrombus were plotted. Stable thrombus formation was defined as the presence of occlusive thrombus at least for 10 min after the initial occlusion.

### 2.7. Statistical Analysis

Prism software (version 3.0) was used for all statistical analyses. The data were given as mean with standard error (SE). Student’s t-test was used to establish statistical significance.

## 3. Results

### 3.1. Arrestin3 Selectively Regulates Agonist-Induced Platelet Aggregation and Secretion

**Figure 1:**
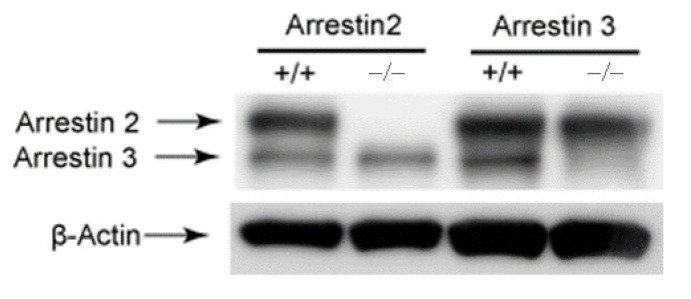

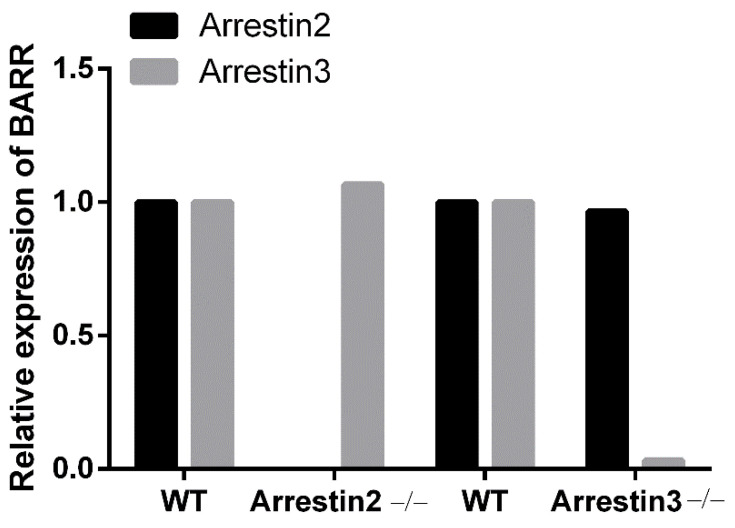
Expression levels of arrestins in platelets. Washed platelets from arrestin2 and arrestin3 +/+ and −/− mice were lysed and probed with anti-arrestin2/3 and anti-β-actin antibodies. Blots are quantified and presented as mean. Statistical analysis was conducted by Student’s *t*-test using Prism software (version 3.0). At least three individual experiments are represented in the Western blots.

**Figure 2:**
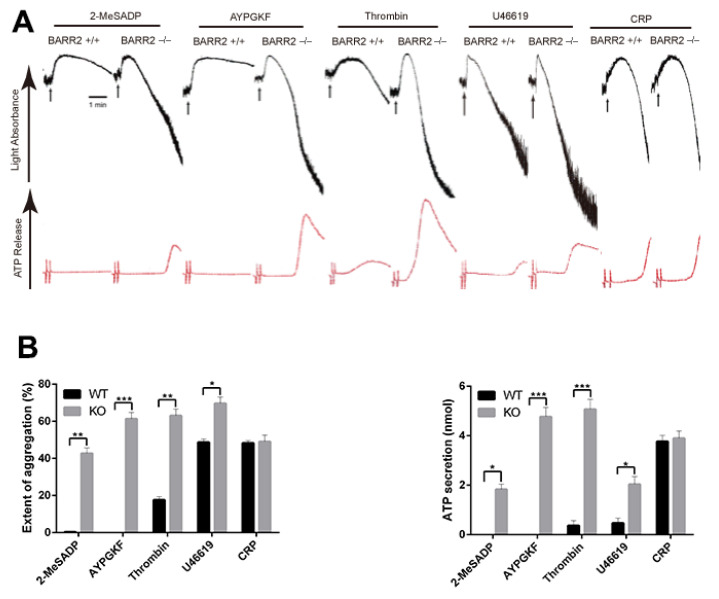

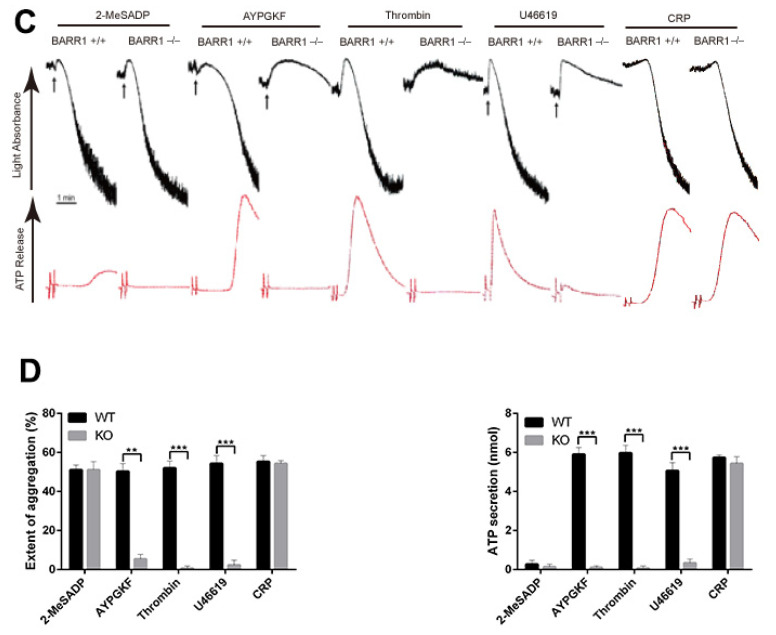
Comparison of agonist-induced platelet aggregation and secretion. (**A**) Washed platelets from arrestin3 −/− (BARR2 −/−) mice and WT (BARR2 +/+) littermates were stimulated with GPCR agonists 30 nM 2-MeSADP, 60 µM AYPGKF, 0.1 U/mL thrombin, and 50 nM U46619 and GPVI agonist 2.5 µg/mL CRP. (**B**) Quantification of extent of aggregation and dense granule secretion from panel A. Data are presented as mean ±SE *, *p* < 0.05; **, *p* < 0.01; ***, *p* < 0.005. (**C**) Washed platelets from arrestin2 −/− (BARR1 −/−) mice and WT (BARR1 +/+) littermates were stimulated with GPCR agonists 50 nM 2-MeSADP, 100 µM AYPGKF, 0.15 U/mL thrombin, and 75 nM U46619 and GPVI agonist 2.5 µg/mL CRP. (**D**) Results quantified from panel C and presented as mean ±SE **, *p* < 0.01; ***, *p* < 0.005. Platelet aggregation (top) and ATP secretion (bottom) were quantified in a lumi-aggregometer. The data are representative of three independent experiments.

To determine the significance of arrestin2 and arrestin3 in platelet function using arrestin −/− mice, we first determined the expression levels of arrestins in mouse platelets. Western blot assay showed that both arrestin2 and arrestin3 are expressed in WT platelets, while only arrestin3 and arrestin2 are detected in arrestin2 and arrestin3 −/− platelets, respectively (Figure 1), verifying the mice genotype. The physiologic effect of arrestins on major agonist-induced platelet aggregation and dense granule secretion were assessed in WT and arrestin-deficient platelets. As shown in Figure 2A, platelet aggregation and corresponding dense granule secretion in response to GPCR agonists, including 2-MeSADP, AYPGKF, thrombin, and U46619, were significantly potentiated in arrestin3-deficient platelets compared to the WT platelets. In contrast, GPVI receptor agonist collagen-related peptide (CRP)-induced platelet aggregation and dense granule secretion were similar in both WT and arrestin3-deficient platelets, indicating that arrestin3 selectively regulates platelet aggregation and secretion in response to GPCR agonists.

Notably, in contrast to arrestin3-deficient platelets, AYPGKF-, thrombin- and U46619-induced platelet aggregation and dense granule secretion were reduced, while 2-MeSADP-induced platelet aggregation and secretion were not affected in arrestin2-deficient platelets compared to the WT platelets (Figure 2C). Similar to arrestin3, arrestin2 had no effect on CRP-induced platelet aggregation and dense granule secretion. These results suggest that arrestin2, unlike arrestin3, does not play any role in PAR-induced GPCR desensitization in platelets.

### 3.2. Arrestin3 Regulates Serotonin- and Epinephrine-Induced Platelet Aggregation

**Figure 3:**
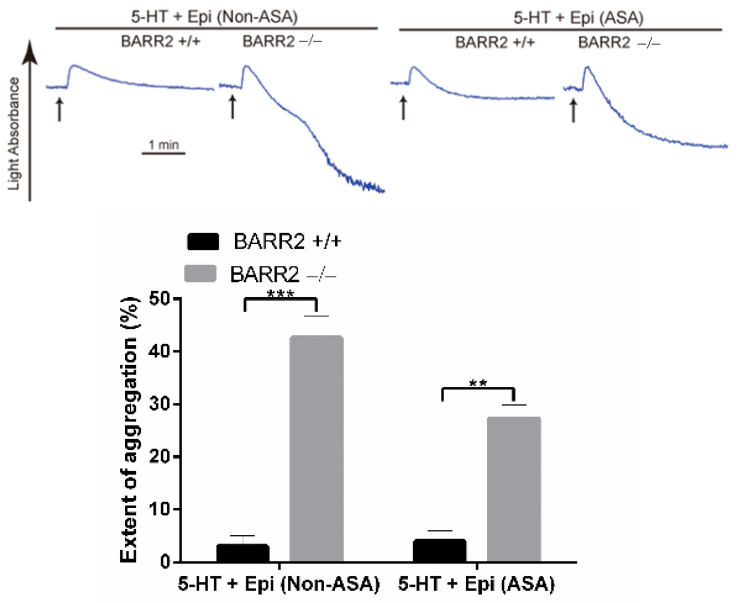
Arrestin3 regulates serotonin- and epinephrine-induced platelet aggregation. Non-aspirin (Non-ASA)- and aspirin (ASA)-treated washed platelets from WT (BARR2 +/+) and arrestin3 −/− (BARR2 −/−) mice were stimulated with 20 µM 5-HT + 100 µM epinephrine, and platelet aggregation was assessed. Data are quantified and presented as mean ±SE **, *p* < 0.01; ***, *p* < 0.005. Three distinct experiments are represented in the tracings.

The co-stimulation of serotonin (5HT) and epinephrine activates the G_q_-coupled 5HT_2A_ receptor and the G_z_-coupled α_2A_ adrenergic receptor, respectively, to induce platelet aggregation that mimics the ADP-induced platelet aggregation mediated by the co-activation of G_q_-coupled P2Y_1_ and G_i_-coupled P2Y_12_ receptors [30]. Recently, we have shown that GRK6 does not control the regulation of the 5HT_2A_ receptor and the α_2A_ adrenergic receptor in platelets, demonstrating that GRK6 selectively regulates GPCR-mediated platelet function through selective GPCR desensitization [31]. In order to assess the involvement of arrestin3 in the differential regulation of GPCRs, we examined platelet aggregation induced by the co-stimulation of serotonin and epinephrine in non-aspirin- and aspirin-treated platelets. Surprisingly, in contrast to GRK6, platelet aggregation induced by both serotonin and epinephrine was significantly potentiated in non-aspirin- and aspirin-treated arrestin3-deficient platelets (Figure 3). This strongly shows that arrestin3 plays a central role in regulating general GPCR signaling.

### 3.3. Arrestin3 Regulates ADP and PAR4 Receptor Desensitization in Platelets

**Figure 4:**
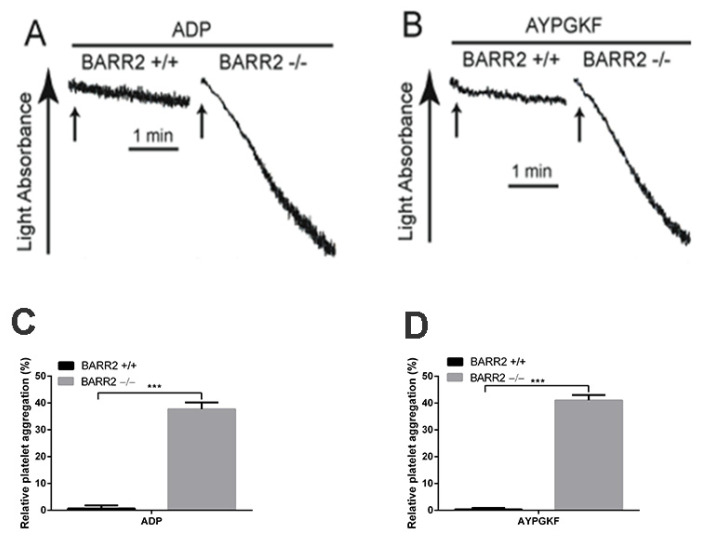
Desensitization of platelet aggregation in response to ADP and AYPGKF in arrestin3-deficient platelets. Washed platelets from WT (BARR2 +/+) and arrestin3 −/− (BARR2 −/−) mice were stimulated with (**A**) 5 µM ADP after 3 min pre-incubation with 5 µM ADP and (**B**) 250 µM AYPGKF after 30 min pre-incubation with 250 µM AYPGKF, and platelet aggregation was assessed. (**C**) Quantification of relative aggregation from panel A. (**D**) Quantification of relative aggregation from panel B. Data are presented as mean ±SE ***, *p* < 0.005. The results are a representation of three independent experiments.

In order to confirm the role of arrestin3 in GPCR desensitization in platelets, we assessed platelet aggregation in response to the re-stimulation of ADP and AYPGKF in WT and arrestin3-deficient platelets. Platelet aggregation was restored in arrestin3-deficient platelets in response to the second challenge of ADP and AYPGKF following pretreatment with these agonists, although re-stimulation with these agonists failed to induce aggregation in WT platelets (Figure 4). These results demonstrate that arrestin3 contributes to P2Y_1_, P2Y_12_, and PAR4 receptor desensitization in platelets.

### 3.4. Potentiation of GPCR-mediated Signaling Events in Arrestin3-Deficient Platelets

**Figure 5:**
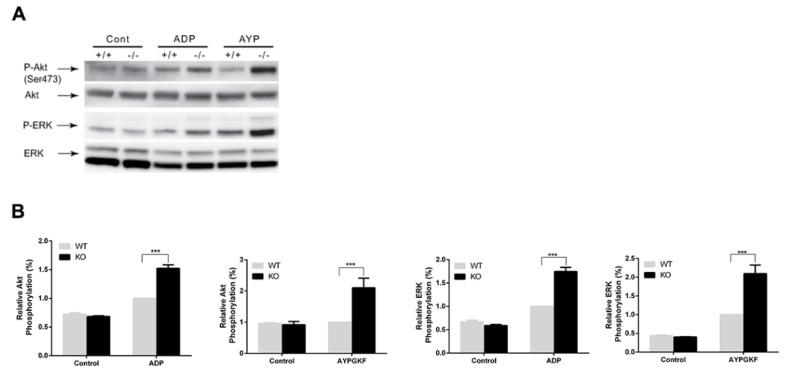
Enhancement of GPCR-mediated signaling events in arrestin3-deficient platelets. (**A**) Platelets from arrestin3 +/+ and −/− mice were stimulated with 50 nM 2-MeSADP and 100 µM AYPGKF for 2 min and probed with anti-phospho-Akt (Ser^473^), anti-phospho-ERK, anti-Akt, or anti-ERK antibodies. (**B**) Data are presented as mean ±SE. ***, *p* < 0.005. Statistical analyses were performed by Student’s t-test using Prism software. Data are a representation of at least three experiments.

To determine the effect of arrestin3 on GPCR-mediated platelet signaling, we stimulated platelets with 2-MeSADP and AYPGK in WT and arrestin3-deficient platelets and compared the phosphorylation of Akt and ERK as described previously [28]. The phosphorylation of Akt and ERK mediated by 2-MeSADP and AYPGKF was potentiated more so in arrestin3-deficient platelets than in WT platelets (Figure 5). These findings suggest that arrestin3 plays a role in regulating GPCR-mediated signaling events in platelets.

### 3.5. Role of Arrestin3 in Regulation of Thrombus Formation In Vivo

**Figure 6:**
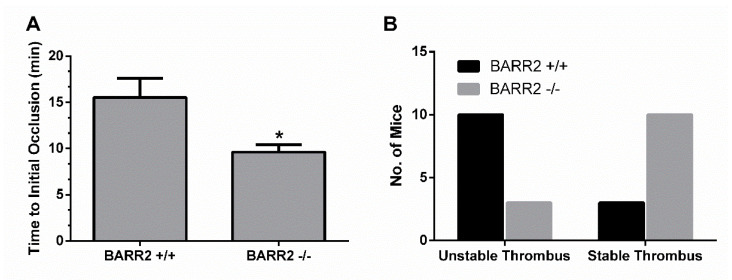
Effect of arrestin3 on enhanced thrombus formation in vivo. To damage the carotid arteries of WT (*n* = 13) and arrestin3 −/− (*n* = 13) mice, 5% FeCl_3_ was used for 90 sec. Following vascular damage, flow rates via the carotid artery were recorded for 30 min with a Doppler flow probe. (**A**) Time to initial occlusion of the carotid artery in WT and arrestin3 −/− mice was recorded. Data are mean ±SE. *, *p* < 0.05. (**B**) Thrombus stability in the carotid artery of WT and arrestin3 −/− mice was determined by counting the number of mice with stable thrombi for at least 10 min.

To determine if arrestin3 has a similar platelet function in vivo, we employed an in vivo thrombosis model using FeCl_3_ injury of the carotid artery [29,32] and measured the time to occlusion and the stability of the thrombus in WT and arrestin3 −/− mice. Initial thrombosis formation was delayed by 6 min in WT mice compared to arrestin3 −/− mice, which formed a stable thrombus within 9 min after the injury, suggesting that arrestin3 −/− mice have enhanced thrombus formation following vascular injury (Figure 6A). In addition, stable thrombus formation was observed in 77% of arrestin3 −/− mice, while only 33% of WT mice showed stable thrombus formation (Figure 6B), indicating enhanced thrombus stability in arrestin3 −/− mice. These findings indicate that arrestin3 is critical for platelet function in vivo.

## 4. Discussion

Platelet activation is important in hemostasis and thrombosis. Platelets can be activated by several agonists, which act through GPCRs to mediate their cellular actions. Similarly, the desensitization mechanism plays a crucial role in switching off receptor-mediated signal transduction pathways and is employed by the majority of GPCRs. Arrestin2 and arrestin3 are widely expressed at relatively similar levels [33] and are confined to GPCRs phosphorylated by GRKs to mediate desensitization and subsequent termination of G protein signaling [34]. Whilst some arrestin family members appear to have substrate specificity, most demonstrate action against a wide spectrum of agonist-occupied receptors in vitro. The arrestins’ involvement has been difficult to pinpoint due to this, as well as due to their widespread tissue expression. Given the pivotal role of GPCR agonists in platelet activation, the functional differences of arrestin2 versus arrestin3 in the regulation of GPCR signaling in platelets are poorly understood. Therefore, we here specifically analyzed the involvement of arrestin3 in the differential regulation of platelet responses mediated by GPCRs using mice lacking arrestin2 and arrestin3.

Although the mechanisms of platelet desensitization to GPCR agonists are not well acknowledged, arrestin isoforms have been demonstrated to be involved in the regulation of particular GPCR desensitization in other cells. Thus, we first investigated whether arrestin2 and arrestin3 had any role in the control of platelet function mediated by GPCRs. The expression levels of arrestin2 and arrestin3 were not altered in arrestin3- and arrestin2-deficient platelets, suggesting that the deletion of one arrestin isoform does not affect other arrestin expressions in platelets. Platelet GPCRs regulate platelet function by coupling to their respective G protein upon agonist stimulation, such as G_q_-coupled P2Y_1_ (ADP), PARs (thrombin), TP receptor (TxA_2_), 5HT (serotonin); G_i_-coupled P2Y_12_ (ADP); and G_z_-coupled α_2A_ adrenergic receptors (epinephrine). In contrast to the investigation conducted by Schaff et al., who reported that arrestin3 deficiency does not alter platelet activation [24], platelet aggregation and dense granule secretion elicited by GPCR agonists, such as 2-MeSADP, AYPGKF, and thrombin, were more potentiated in the arrestin3-deficient platelets than in the WT platelets in our study. Supporting our study, a very recent study also reported the negative regulatory role of arrestin3 downstream of PAR4- and P2Y_12_-mediated signaling in platelets [25]. At present, it is not known whether arrestins regulate TxA_2_-induced aggregation in platelets. Interestingly, we found that TxA_2_ analog U46619-induced platelet aggregation and dense granule secretion were significantly potentiated in arrestin3-deficient platelets, suggesting its negative regulatory role in TxA2-induced platelet response. Moreover, we observed that in the arrestin3-deficient platelets, GPVI agonist CRP-induced platelet aggregation and secretion were not altered. Taken together, our study suggests that arrestin3 regulates P2Y_1_-, P2Y_12_-, TP-, and PAR-mediated signaling in platelets, while it does not regulate non-GPCR-mediated platelet function.

It is not clear about the significance of arrestin2 in the modulation of platelet function. It has been demonstrated that both non-visual arrestin family members negatively regulate GPCR-mediated signaling [35,36]. Previously, it was shown that arrestin2 does not affect platelet function [24]. By contrast, compared to WT, we observed that GPCR agonist-induced platelet aggregation and dense granule secretion were significantly reduced in arrestin2-deficient platelets. Initially recognized as a moderator of GPCR desensitization, arrestins are now understood to be adaptor proteins that transmit signals to a variety of effector pathways. It has been suggested that arrestin2 can act as a scaffold to recruit PI3K to the PAR4 receptor for thrombin [23]. Thus, the inhibition of AYPGKF- and thrombin-induced platelet aggregation and secretion in arrestin2-deficient platelets might be due to the positive regulatory role of arrestin2 in PAR4-mediated PI3K/Akt signaling, which is well established for its role in platelet activation. Furthermore, it was also demonstrated that ADP-induced Akt and fibrinogen binding were unaffected in arrestin2-deficient platelets [23], which indicates why ADP-induced platelet aggregation and secretion were not altered in arrestin2-deficient platelets in our study. Collectively, our observations show that arrestin isoforms selectively and differentially regulate GPCR-mediated platelet response; unlike arrestin3, arrestin2 positively modulates PAR- and TP receptor-induced platelet aggregation and secretion; arrestin3 functions in the termination of GPCR signaling in platelets, and arrestin2 may not be involved in the desensitization of GPCRs in platelets.

In our recent study, we found that GRK6 negatively regulated ADP-induced platelet aggregation, while it did not have any role in serotonin- and epinephrine-induced platelet aggregation. In contrast, intriguingly in our study, we observed that platelet aggregation induced by the co-stimulation of serotonin and epinephrine was potentiated in the arrestin3-deficient platelets. We also checked the effect of arrestin3 in aspirin-treated platelets, and our data indicated that the potentiation of aggregation in the arrestin3-deficient platelets is not due to the effect of arrestin3 on the positive feedback effect of generated TxA_2_. Importantly, these results suggest that arrestin3 plays a role in the regulation of α_2A_ and 5HT_2A_ receptors in platelets. The mechanism of GPCR-mediated signaling by arrestin is downstream of GRK’s kinase activity, as arrestins are recruited to the receptor after GRKs phosphorylate ligand-activated GPCRs and ultimately terminate and internalize the receptor [37]. Therefore, the effect of arrestin on serotonin- and epinephrine-mediated aggregation in platelets may be due to the kinase activity of GRK isoforms other than GRK6. Together, our data indicate that in contrast to the involvement of GRK6 in selective GPCR function, arrestin3 plays a central role in general GPCR signaling in platelets.

The agonist-mediated desensitization of many GPCRs is regulated by GRKs and arrestins. It is possible that GRKs and arrestins regulate GPCR desensitization in platelets, thus preventing prolonged or inappropriate receptor-mediated signaling. Considering the importance of GPCR-mediated signaling in platelets, the underlying mechanism involved in receptor desensitization in platelets to various agonists remains largely unknown. Hence, a comprehensive understanding of the mechanisms involved in the regulation of receptor desensitization and internalization to platelet agonists is of considerable importance for the design of improved therapeutic strategies in the treatment of thrombotic disease. Similar to our earlier work where we had shown that the GRK6 isoform induces ADP (both P2Y_1_ and P2Y_12_) and PAR4 receptor desensitization in platelets [31], we observed that ADP- and AYPGKF-induced platelet aggregation were restored with the re-stimulation of platelets with these agonists in arrestin3-deficient platelets, demonstrating that arrestin3 also has a distinct role in regulating GPCR desensitization in platelets. These data confirm that arrestin3 contributes to the desensitization of ADP and PAR4 receptors in platelets.

In contrast to our results showing that PAR4 desensitization was markedly impaired in arrestin3-deficient platelets, it has been shown that PAR1 desensitization is markedly diminished in mouse embryonic fibroblasts (MEFs) deficient in only arrestin2 compared with arrestin3-deficient or WT cells [17], suggesting that arrestin2 is the crucial mediator of PAR1 desensitization. Since murine platelets do not contain PAR1, it is not possible to identify the role of arrestin3 in PAR1 desensitization in platelets. Our study clearly shows the difference in the role of arrestin2 and arrestin3 in regulating the signaling cascade by GPCRs in platelets compared to other cell types, and arrestin2 may not be involved in PAR1 desensitization in platelets.

G protein-mediated signaling by GPCRs leads to the activation and phosphorylation of Akt, ERK, and PKCδ, which are important for the promotion and enhancement of platelet aggregation [38,39]. Since the classical function of arrestins is to terminate these GPCR-mediated signaling events, we activated the platelets from arrestin3 −/− mice and their matching WT mice with ADP and AYPGKF and compared the phosphorylation of downstream molecules of GPCR signaling. We found that the phosphorylation events were significantly potentiated in arrestin3-deficient platelets, suggesting that arrestin3 potentiates ADP and PAR4 receptor-mediated signaling events through GPCR desensitization in platelets. Consistent with in vitro data, we found that FeCl_3_ injury-induced in vivo thrombosis models resulted in increased hemostatic function through enhanced thrombus growth and stability in arrestin3-deficient mice. Moreover, we measured tail bleeding time in WT and arrestin3 −/− mice and found that arrestin3 −/− needed 37 sec for complete blockade of tail bleeding compared to 56 sec needed by WT mice, suggesting the role of arrestin3 in the regulation of hemostatic function in vivo (data not shown). This physiological effect might be the result of arrestin3-mediated termination of overall platelet response, and our study confirmed the important role of arrestin3 in platelet function in vivo.

In conclusion, we demonstrate that arrestin3, not arrestin2, plays a central role in regulating GPCR-mediated platelet functional responses and thrombus formation in vivo through general GPCR desensitization.
